# The major role of *Listeria monocytogenes* folic acid metabolism during infection is the generation of N-formylmethionine

**DOI:** 10.1128/mbio.01074-23

**Published:** 2023-09-11

**Authors:** Ying Feng, Shannon K. Chang, Daniel A. Portnoy

**Affiliations:** 1 Department of Molecular and Cell Biology, University of California, Berkeley, California, USA; 2 Department of Plant and Microbial Biology, University of California, Berkeley, California, USA; University of Illinois Chicago, Chicago, Illinois, USA

**Keywords:** folic acid, *Listeria monocytogenes*, N-formylmethionine, purine, tetrahydrofolate

## Abstract

Folic acid and its derivatives (folates) play central roles in one-carbon metabolism, necessary for the synthesis of purines, pyrimidines, and some amino acids. Antifolate drugs are widely used as broad-spectrum antibiotics for bacterial infections, including listeriosis, a disease caused by the facultative intracellular pathogen *Listeria monocytogenes*. However, folate-derived metabolites required during bacterial infection are poorly understood. Here, we report that *L. monocytogenes* encodes two enzymes, methylenetetrahydrofolate dehydrogenase/methenyltetrahydrofolate cyclohydrolase (FolD) and formyltetrahydrofolate synthetase (Fhs), that catalyze the formation of N10-formyltetrahydrofolate, a critical intermediate in folate metabolism. N10-formyltetrahydrofolate is an essential carbon donor for biosynthesis of purines and N-formylmethionine, the amino acid used during initiation of bacteria translation. While *L. monocytogenes* mutants lacking Fhs had no observable defects and mutants lacking FolD had moderate virulence defects, mutants lacking both were highly attenuated in mice, especially in the liver. We compared the growth and virulence of mutants that were unable to synthesize folates with mutants unable to generate folate downstream products, including purines, thymidine, and N-formylmethionine. Mutants unable to synthesize N-formylmethionine behaved almost identically to FolD/Fhs double mutants during growth in broth or macrophages, but most importantly, were similarly attenuated to mutants unable to make folates, both of which had approximately 4-log_10_ less colony-forming units in the livers compared with wild-type *L. monocytogenes*. The purine auxotrophic mutants were only 1.5-log_10_ less virulent, and the thymidine mutants were fully virulent in the mouse infection model. These results strongly suggest that the main role of *L. monocytogenes* folates during infection is the generation of N-formylmethionine.

**Importance:**

Folic acid is an essential vitamin for bacteria, plants, and animals. The lack of folic acid leads to various consequences such as a shortage of amino acids and nucleotides that are fundamental building blocks for life. Though antifolate drugs are widely used for antimicrobial treatments, the underlying mechanism of bacterial folate deficiency during infection is unclear. This study compares the requirements of different folic acid end-products during the infection of *Listeria monocytogenes*, a facultative intracellular pathogen of animals and humans. The results reveal the critical importance of N-formylmethionine, the amino acid used by bacteria to initiate protein synthesis. This work extends the current understanding of folic acid metabolism in pathogens and potentially provides new insights into antifolate drug development in the future.

## INTRODUCTION

Folic acid is an essential B vitamin in all three domains of life that is synthesized by most bacteria, some archaea, plants, but not mammals (1 – 4). The bioactive derivatives of folic acid, tetrahydrofolates (THFs), play central roles in one-carbon (1C) metabolism by donating carbon groups necessary for the synthesis of purines, pyrimidines, amino acids, and N-formylmethionine (fMet), the first amino acid used during protein translation in bacteria and mitochondria (5, 6). Synthesis of THFs and their downstream metabolites are proven antibiotic targets (7 – 9). Sulfa drugs target the folate precursor para-aminobenzoic acid (PABA) biosynthesis, while trimethoprim targets dihydrofolate reductase and is generally used together as broad-spectrum antibiotics for many bacterial infections, including listeriosis, a disease caused by a facultative intracellular pathogen *Listeria monocytogenes* (10, 11). Despite the importance of current and new antifolate drugs, and the extensive studies of folic acid metabolism, especially in humans, the functions of folic acid metabolism during infection by intracellular pathogens remain poorly understood.

**Fig 1:**
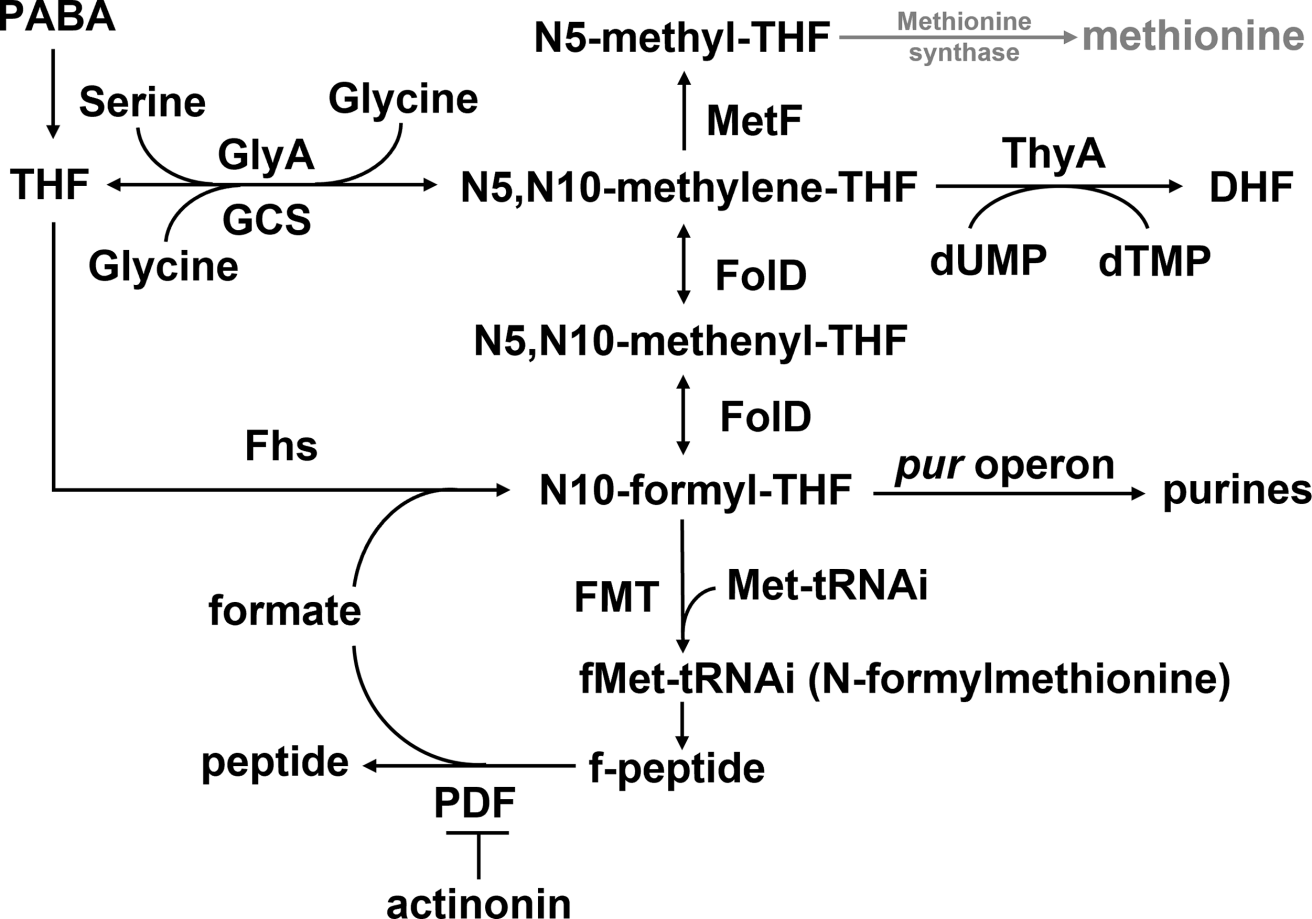
Simplified view of folate metabolism in *L. monocytogenes*. The pathway involves tetrahydrofolates carrying one-carbon units, and N-formylmethionine, purine, and thymidine as key products. Gray, enzymes and products that are absent in *L. monocytogenes* 10403S. dTMP, deoxythymidine monophosphate; dUMP, deoxyuridine monophosphate; FolD, methylenetetrahydrofolate dehydrogenase/methenyltetrahydrofolate cyclohydrolase; GCS, glycine cleavage system; GlyA, serine hydroxymethyltransferase; MetF, 5,10-methylene-THF reductase; THP, tetrahydrofolate; ThyA, thymidylate synthase.

*L. monocytogenes* is a rapidly growing, Gram-positive facultative intracellular pathogen that can cause serious, sometimes fatal, disease following ingestion of contaminated food in a wide range of animals, including immunocompromised and pregnant humans (12 – 14). *L. monocytogenes* also serves as a highly tractable model organism for studying intracellular pathogens and cell-mediated immunity (12, 15). In a survey to identify *L. monocytogenes* transposon mutants that formed small plaques in monolayers of tissue culture cells, we identified two genes that encode enzymes in folic acid metabolism (16). Mutants in *pabBC*, which encodes two enzymes that catalyze the biosynthesis of the folic acid precursor, PABA, were severely attenuated for *L. monocytogenes* virulence in tissue culture and mouse infection models (17), although the precise reasons for its virulence were not established. Additionally, we identified *folD*, which encodes bifunctional methylenetetrahydrofolate dehydrogenase/methenyltetrahydrofolate cyclohydrolase, that catalyzes the reversible conversion of two major 1C-carrying folates, N5,N10-methylene-THF and N10-formyl-THF. The 1C group of N5,N10-methylene-THF is used to generate serine, glycine, and deoxythymidine monophosphate (dTMP) while that of N10-formyl-THF is used to synthesize purines and fMet (Fig. 1). FolD is essential in many bacteria, although some facultative anaerobes, including *L. monocytogenes*, can also synthesize N10-formyl-THF using formyltetrahydrofolate synthetase (formate tetrahydrofolate ligase, Fhs) (Fig. 1) (18). In bacteria and mitochondria, methionyl-tRNA formyltransferase (FMT) transfers the formyl-group from N10-formyl-THF to a methionine bound to the initiator tRNA which in turn promotes the initiation of translation (19, 20).

In this study, we sought to explore the functional mechanisms underlying the requirement of folate metabolism during *L. monocytogenes* infection. We characterized the roles of FolD and Fhs during bacterial growth in media, tissue culture, and animal infection models. FolD played a dominant role in making N10-formyl-THF and was critical to *L. monocytogenes* aerobic growth and pathogenesis, although mutants lacking both FolD and Fhs were much more highly attenuated. By comparing the phenotypes of mutants in different steps in folic acid metabolism, we concluded that fMet was the most critical folate end-product required during infection *in vivo*.

## RESULTS

### Characterization of *folD* and *fhs* mutants in media and in tissue culture models of infection

**Fig 2:**
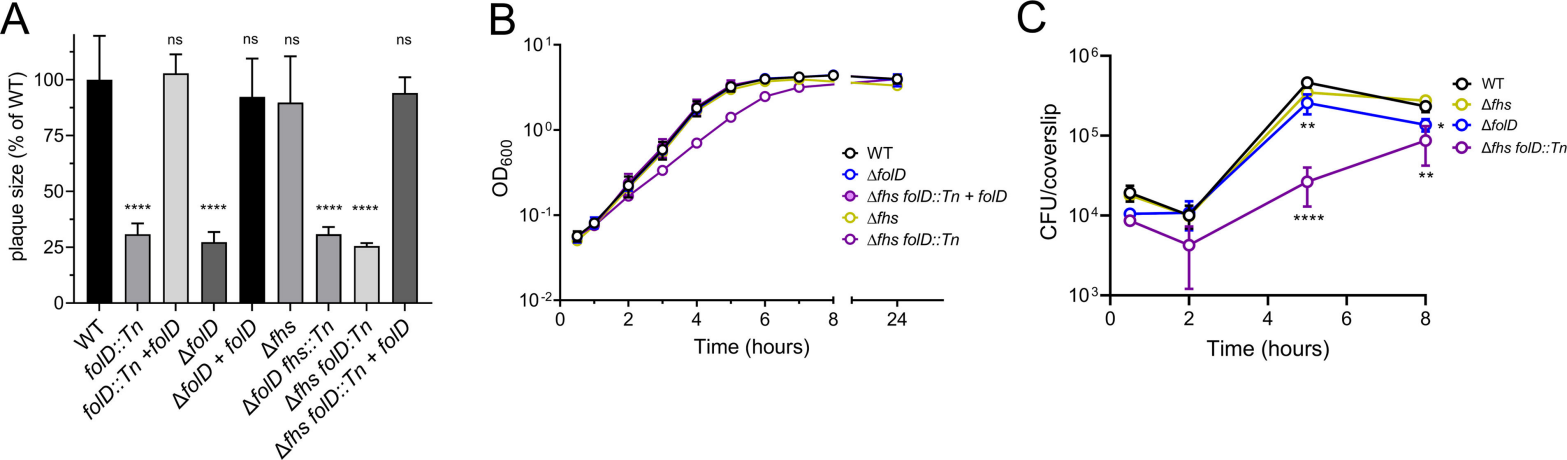
N10-formyl-THF is required for optimal growth in broth and host cells. (**A**) Plaque formation of indicated strains in L2 cells measured 3-d post-infection as a percentage of wild type. (**B**) Broth growth curve in brain heart infusion medium cultured at 37°C with agitation. Growth of *L. monocytogenes* strains was measured spectrophotometrically. (**C**) Intracellular growth in bone marrow-derived macrophages. BMMs were infected at a multiplicity of infection (MOI) of 0.25 for 30 min. Extracellular growth of bacteria was prevented by adding 50 µg/mL gentamicin at 1-h post-infection. Growth was enumerated by plating the colony-forming units (CFUs) at indicated time points. (**A–C**) Data are mean ± SD. Three independent experiments were combined. One-way analysis of variance (ANOVA), multiple comparisons with WT; ns, not significant; *P < 0.05; **P < 0.01; ****P < 0.0001.

In an effort to understand the role of *L. monocytogenes* folic acid metabolism during infection, we characterized a previously identified transposon insertion mutant in *folD* (*folD::Tn*), which encodes a central enzyme of folic acid metabolism (Fig. 1), that catalyzes the formation of N10-formyl-THF, a bioactive folate that donates carbon groups essential for both purines and N-formylmethionine synthesis. We noted that *L. monocytogenes* encodes another enzyme, formate tetrahydrofolate ligase (Fhs), which also catalyzes the formation of N10-formyl-THF. We constructed in-frame deletions in *L. monocytogenes folD* (Δ*folD*), in *fhs* (Δ*fhs*), and double mutants lacking both (Δ*fhs folD::Tn* and Δ*folD fhs::Tn*). Consistent with our previous findings characterizing the *folD::Tn* mutant (16), the Δ*folD* deletion mutant formed very small plaques in monolayers of murine L2 fibroblasts (Fig. 2A). The Δ*fhs* strain formed plaques indistinguishable from wild-type (WT), and the double mutants Δ*fhs folD::Tn* formed plaques similar to those formed by Δ*folD* (Fig. 2A), indicating that FolD plays the major role in generating N10-formyl-THF during host cell infection *in vitro*. Neither single mutant exhibited a growth defect in rich media, while the double mutants grew significantly slower compared to WT, with a 1.8-fold increased doubling time (Fig. 2B; Table S1). The growth defect observed in strains lacking *folD* and *fhs* was fully rescued by complementing with the *folD* gene expressed from a *pHyper* promoter (21) (Fig. 2A and B). We also examined the requirements of *folD* and *fhs* during infection of mouse bone marrow-derived macrophages (BMMs). Although the Δ*folD* strain had no defect when cultured in broth, it showed a small but significant defect at 5- and 8-h post-infection in BMMs (Fig. 2C). Notably, the intracellular growth rate of the Δ*fhs folD::Tn* double mutant was highly impaired, although it was able to grow until it reached a similar terminal OD_600_ as WT in broth (Fig. 2B and C; Table S1). Taken together, these data demonstrated that N10-formyl-THF is critical for *L. monocytogenes* growth and host cell infection. Although FolD is the dominant enzyme required for the synthesis of N10-formyl-THF, Fhs plays a measurable role in the absence of FolD.

### Characterization of *folD* and *fhs* mutants in mice

**Fig 3:**
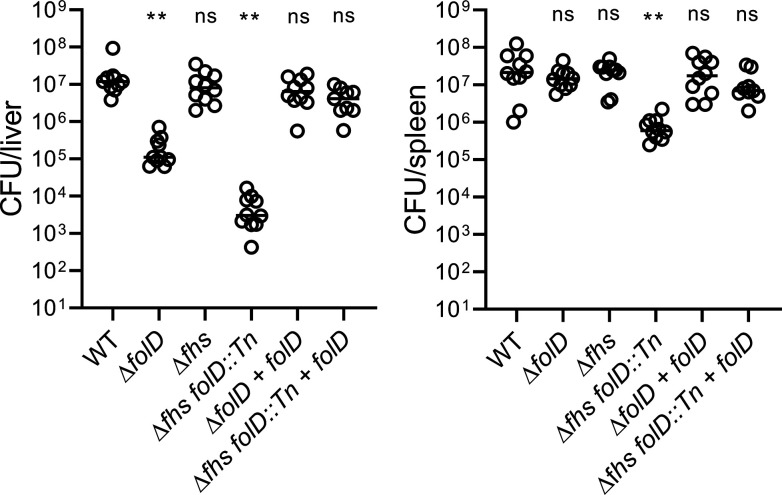
N10-formyl-THF is critical for virulence in mice. Eight-wk-old CD-1 mice (Charles River) were infected intravenously with 1 × 10^5^ CFUs of indicated strains. Bacterial burdens in livers and spleens were measured 48-h post-infection by plating homogenized organs. Each circle represents an individual mouse. Lines present medians. Two biological repeats were combined with a total of 8–10 mice per strain. One-way ANOVA, multiple comparisons with WT; ns, not significant; **P < 0.01.

To reveal the requirements of N10-formyl-THF during *in vivo* infection, CD-1 mice were infected intravenously, and bacterial burdens were determined in the livers and spleens 48-h post-infection. The Δ*folD* mutant exhibited an organ-specific defect that presented as a 2-log_10_ virulence attenuation in the livers compared with WT and the complemented strains but showed no difference in colony-forming units (CFUs) in the spleens (Fig. 3). A main difference between these two organs is that in the spleen, *L. monocytogenes* grows in phagocytic cells, while much of the growth in the liver is in hepatocytes (22). To address if the liver-specific defect was due to poor growth in hepatocytes, the *folD::Tn* transposon was transduced into a strain lacking ActA and InlB, which prevents the bacteria from spreading into hepatocytes or entering via InlB-induced internalization (23). In the Δ*actA* Δ*inlB* background, lack of *folD* still resulted in attenuation in the livers (Fig. S1A). In addition, *folD* played a negligible role in plaque formation in a murine hepatocyte cell line TIB-73 (Fig. S1B). These data suggested that the organ-specific defect of mutants lacking *folD* was unlikely attributed to the infection of hepatocytes.

*L. monocytogenes* virulence factor expression is controlled by the transcription factor PrfA (24, 25), and the biosynthesis of PABA, the folic acid precursor, is activated by PrfA (17). We wondered if the virulence defect of a *folD* mutant was due to lack of PrfA activation or if PrfA activation could compensate the defect of a *folD* mutant. To evaluate the role of PrfA activation, we introduced the Δ*folD* deletion mutation into a PrfA* strain where PrfA has a mutation that locks it in its active conformation (25). The PrfA* Δ*folD* double mutant remained attenuated in the livers (Fig. S2), indicating that the defect of Δ*folD* was probably related to poor growth rather than loss of virulence gene activation.

Mutants lacking both *folD* and *fhs* (Δ*fhs folD::Tn*) were severely attenuated *in vivo*, with approximately 4-log_10_ fewer CFUs in infected livers compared with WT and a 1.5-log_10_ defect in the spleens (Fig. 3). Complementation of Δ*fhs folD::Tn* with *folD* fully restored virulence (Fig. 3), again highlighting the dominant role of *folD* during infection. As observed in broth and BMMs, loss of *fhs* had no observable phenotype in the presence of *folD* but had a synergistic virulence defect when combined with a *folD* mutation during *in vivo* infection. We speculated that the role of Fhs during aerobic growth and during infection was limited due to the absence of its substrate formate, which is produced mostly during fermentation (26). Indeed, the growth defect of a Δ*folD* mutant growing aerobically in a chemically defined medium was completely restored if supplemented with formate, while restoration by formate was not observed in the absence of *fhs* as shown by the *Δfhs folD::Tn* strain (Fig. S3).

### Genetic screen to identify mutations that rescue the virulence defect of a ∆*folD* mutant and the role of purine biosynthesis during infection

We next sought to understand the mechanisms underlying the virulence defect of *L. monocytogenes* deficient in making N10-formyl-THF. THFs are carbon donors for various molecules, thus perturbations in folate metabolism could result in pleiotropic phenotypes (5, 6, 27). We showed that N10-formyl-THF made by FolD and Fhs was critical for *L. monocytogenes* growth and pathogenesis (Fig. 2 and 3), but it was unclear which factor(s) led to the virulence defect of the N10-formyl-THF deficient strains. To address this question, we generated a transposon library in a *folD* mutant background and screened for mutants that formed larger plaques in tissue culture. A total of 20 insertion mutants representing eight genes were identified that displayed increased plaque size from over 35,000 screened. Candidate suppressors were confirmed by transducing single insertions into WT and Δ*folD* background (Table S2).

**Fig 4:**
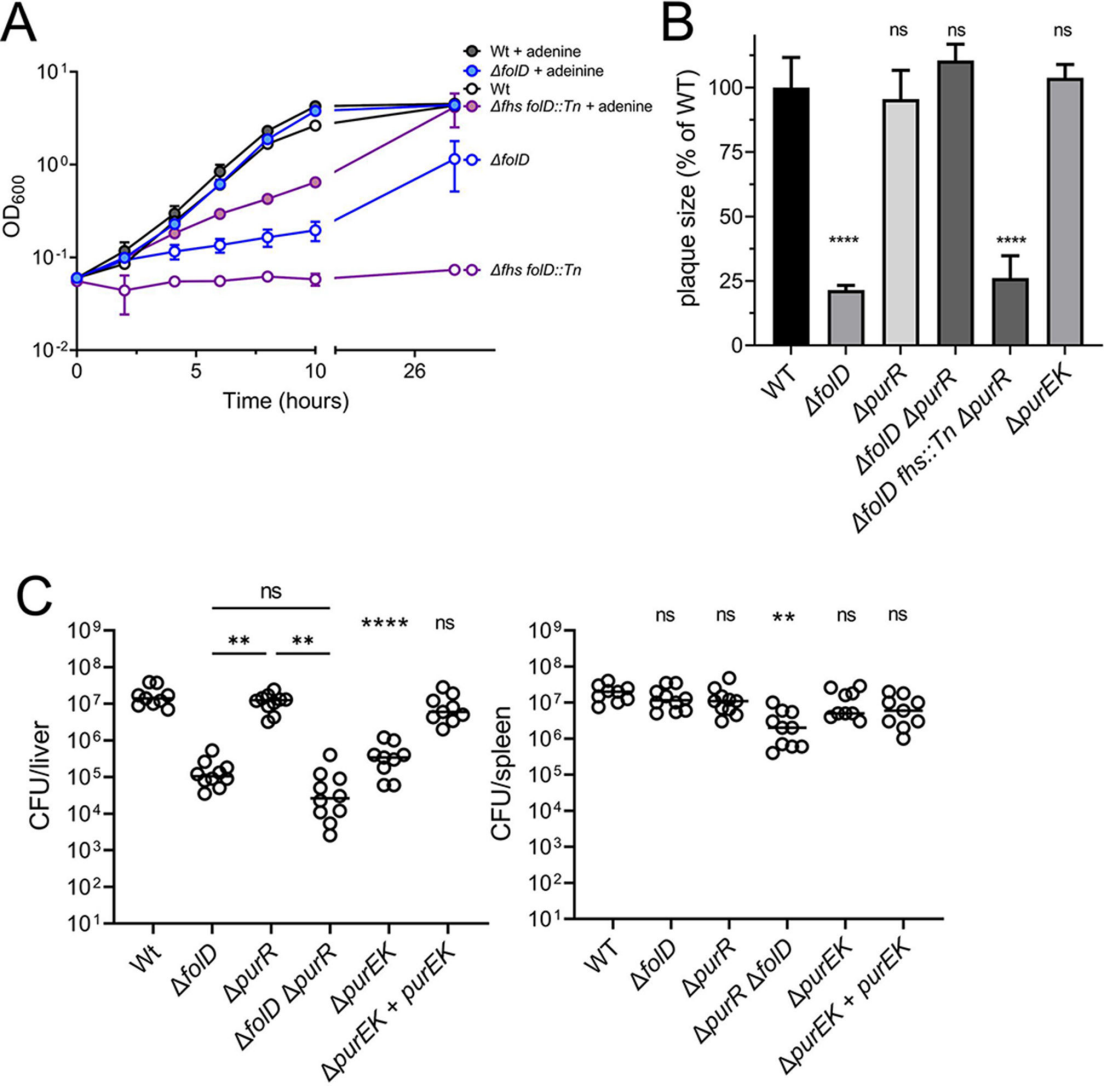
De-repression of PurR restores plaque forming of Δ*folD* but not virulence in mice. (**A**) Broth growth curve in synthetic medium supplemented with adenine. Strains were cultured in the *Listeria* synthetic medium with or without 1 mM adenine at 37°C. Growth was measured spectrophotometrically. (**B**) Plaque formation of indicated strains in L2 cells. Plaques were measured on the third day after infection and presented as a percentage of wild type. Three independent experiments were combined for (A) and (B). Data are mean ± SD. One-way ANOVA, multiple comparisons with WT; ns, not significant; ****P < 0.0001. (**C**) Virulence of indicated *L. monocytogenes* strains presented by CFUs in infected mouse livers and spleens. Two biological repeats were combined with a total of 10 mice per strain. Lines present medians. One-way ANOVA, multiple comparisons with WT control indicated on top of each strain, comparisons among the three mutant strains indicated by lines; ns, not significant; **P < 0.01; ****P < 0.0001.

Insertions disrupting *purR* led to the largest plaque restoration of Δ*folD* (Table S2). The *purR* gene encodes a transcriptional repressor that controls gene expression in response to the availability of purines (28 – 30). The major role of PurR is to repress the transcription of genes in the *pur* operon that encodes enzymes for the *de novo* synthesis of purines, although it represses other genes as well (28, 30 – 32). N10-formyl-THF is used as a cofactor for two *pur*-encoded enzymes that each donates a carbon unit during the construction of the purine ring. We hypothesized that purine shortage might lead to growth and virulence defects during N10-formyl-THF limitation, and de-repression of purine synthetic genes by the *purR* mutation replenished purine levels and consequently restored the growth and virulence of Δ*folD*. As shown in Fig. S3, the growth of Δ*folD* was greatly impaired in chemically defined media without N10-formyl-THF end-products. Adding exogenous purines into the media fully restored the growth of Δ*folD* and partially rescued the Δ*fhs folD::Tn* strain as well (Fig. 4A). A *purR* in-frame deletion was generated in WT and Δ*folD* backgrounds, and consistent with the transposon mutants, the Δ*folD* Δ*purR* mutants formed WT plaques (Fig. 4B). However, the *purR* mutation did not rescue plaque formation in a strain lacking *folD* and *fhs*, or the virulence of Δ*folD* in mice (Fig. 4B and C).

**Fig 5:**
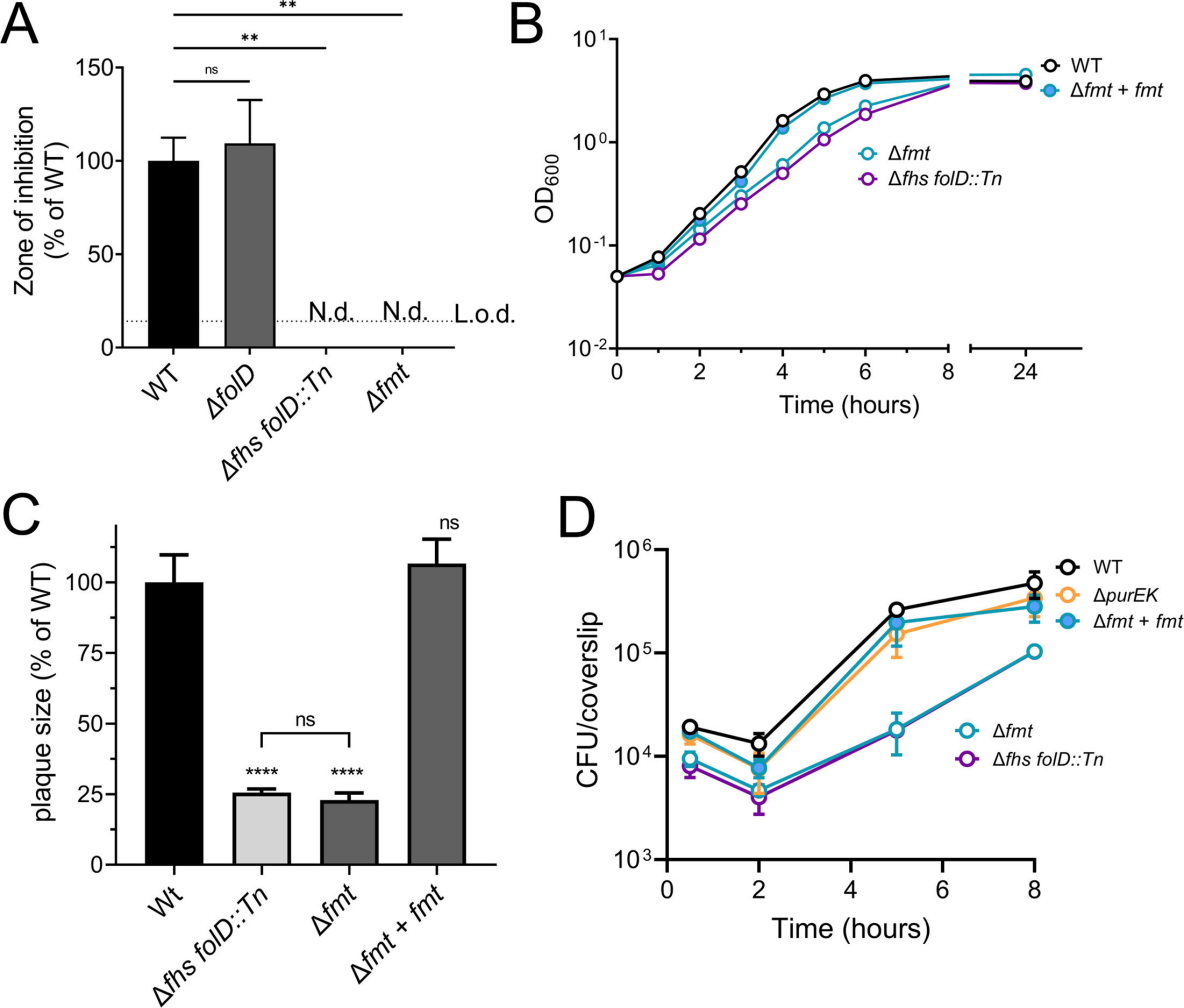
Lack of fMet results in defects in broth growth and host cell infection. (**A**) Antibiotic sensitivity measured by disk diffusion containing 500 µg actinonin on brain heart infusion (BHI)-agar. L.o.d., limit of detection; N.d., not detectable. (**B**) Broth growth curve of indicated strains grown in BHI media at 37°C with agitation. (**C**) Plaque formation in murine fibroblast L2 cells measured 3-d post-infection of the indicated strains as a percentage of WT. (**D**) Intracellular growth in BMMs. Host cells were infected with indicated strains for 30 min at an MOI of 0.25. Antibiotic gentamicin was added to prevent extracellular bacteria at 1-h post-infection. (**A–D**) Three independent experiments were combined. Data are mean ± SD. One-way ANOVA, multiple comparisons with WT; ns, not significant; **P < 0.01; ****P < 0.0001.

In order to directly evaluate the role of *de novo* purine biosynthesis, we generated a purine auxotrophic strain introducing an in-frame deletion of *purE* and *purK* (Δ*purEK*), the first two genes in the *pur* operon. As expected, the Δ*purEK* strain failed to grow in media lacking purines (Fig. S4), but it grew like WT in both the plaque assay and in BMMs (Fig. 4B and Fig. 5D). However, the Δ*purEK* mutant did have an approximately 1.5-log_10_ virulence defect in mouse livers, although it was much less attenuated than a *folD/*fhs double mutant (Fig. 3 and Fig. 4C). Therefore, it is reasonable to conclude that N10-formyl-THF does contribute to purine biosynthesis during *in vivo* infection, and purine insufficiency is partially responsible for the severe attenuation of strains lacking FolD and Fhs.

### Comparison of mutants lacking different steps of the folic acid cycle suggests a critical role of N-formylmethionine during infection

**Fig 6:**
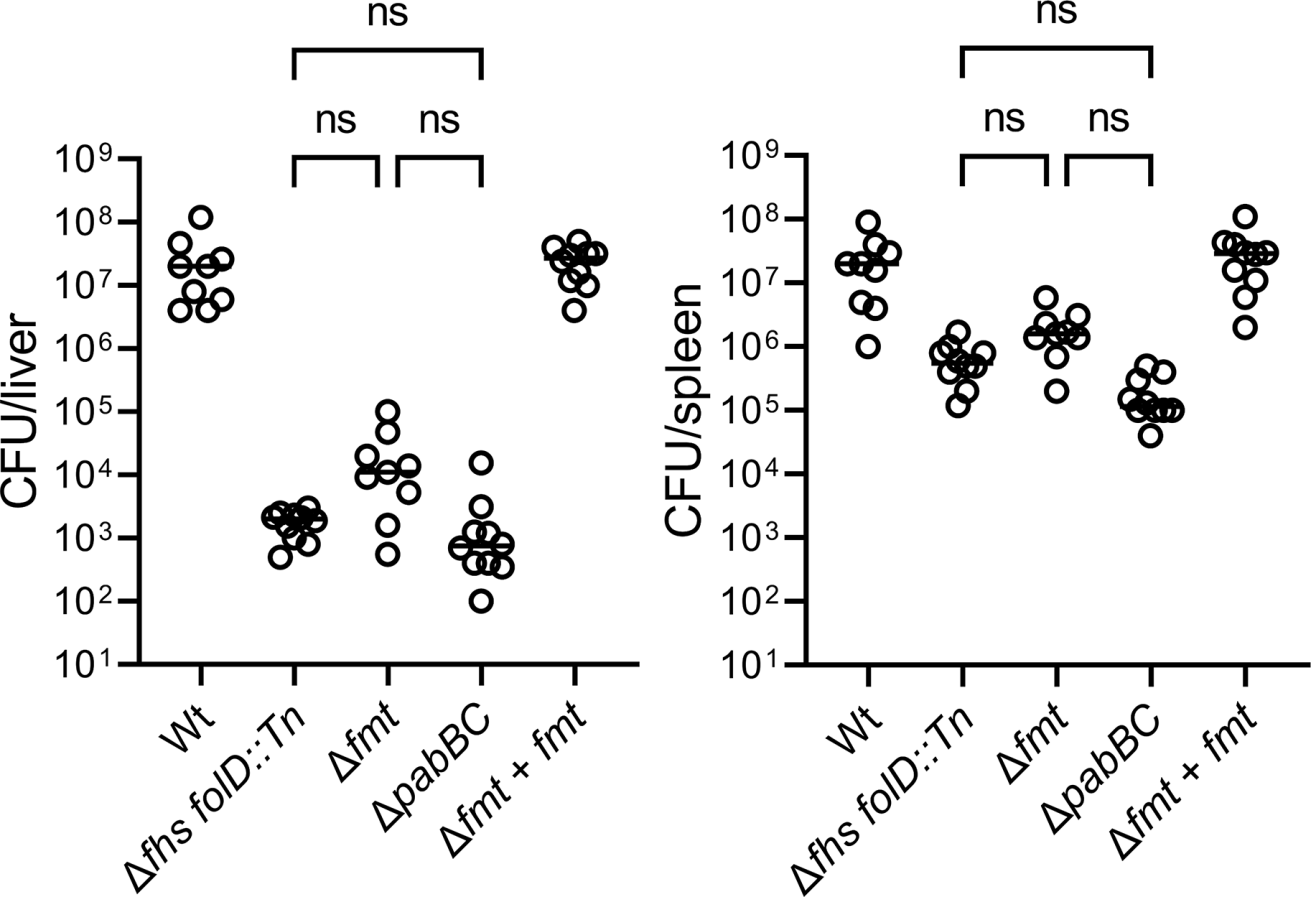
Strains that fail to make fMet are similarly attenuated *in vivo*. CD-1 mice (*n* = 10) were infected with 1 × 10^5^ CFUs of indicated *L. monocytogenes* strains. Bacteria CFUs in mouse livers and spleens were enumerated 2-d post-infection. One-way ANOVA, multiple comparisons among the three mutant strains were indicated; ns, not significant.

In addition to purine biosynthesis, the other one-carbon recipient of N10-formyl-THF is methionine bound to the initiation tRNA (Met-tRNA^i^), catalyzed by methionyl-tRNA formyltransferase (FMT) (Fig. 1). Although *fmt* is essential in some bacteria, it has been mutated in many bacteria, including *L. monocytogenes* (33 – 39). Accordingly, we constructed a strain lacking formylated Met-tRNA^i^ (fMet-tRNA^i^) by in-frame deletion of *fmt* (Δ*fmt*). Removal of the formyl-group from nascent peptides by peptide deformylase (PDF) is essential but is dispensable in strains lacking N-formylmethionine (i.e., fMet-tRNA^i^). Mutants lacking N-formylmethionine are resistant to actinonin, an antibiotic targeting PDF (33). We tested actinonin sensitivity of Δ*fhs folD::Tn* and Δ*fmt* in a disk-diffusion assay and found that both strains were resistant to actinonin (Fig. 5A), strongly suggesting that N-formylmethionine was not being synthesized. We then compared the Δ*fhs folD::Tn* and Δ*fm*t mutants in various assays, and strikingly, the Δ*fmt* strain behaved almost identical to Δ*fhs folD::Tn* in broth growth, plaque formation, growth in BMMs, and virulence in mice (Fig. 5B through D; Fig. 6). The defects observed in the mutants lacking *fmt* were fully rescued by complementation with the *fmt* gene (Fig. 5B through D; Fig. 6).

We next sought to evaluate the relative importance of N-formylmethionine compared with other folate end-products. In addition to purines and N-formylmethionine, the carbon units carried by different folate molecules can be used in the synthesis of serine, glycine, and dTMP, depending on the needs of bacteria (Fig. 1). Previous ^13^C-isotopologue profiling demonstrated that *L. monocytogenes* imports glycine and serine from the host cell cytoplasm (40). Methionine is also a folate product in many species, but *L. monocytogenes* 10403S lacks methionine synthase (Fig. 1) (41, 42), so it was not addressed here. We examined the requirement of thymidine during infection by using a mutant lacking *thyA* (43), which encodes the enzyme transferring the carbon unit from N5,N10-methylene-THF to produce dTMP (Fig. 1). The Δ*thyA* mutants do not grow in BMMs (Fig. S5A) but were fully virulent in the mouse infection model (Fig. S5B), suggesting that generation of dTMP from folates is not necessary during *L. monocytogenes in vivo* infection.

Finally, we compared the virulence defect of an *L. monocytogenes* strain unable to synthesize PABA (Δ*pabBC*) and, therefore, lacks folate synthesis. The folate biosynthesis defective strain, Δ*pabBC*, is severely attenuated *in vivo*, and purine supplementation partially replenished its growth in defined media (17). In the mouse infection model, the Δ*pabBC* and Δ*fhs folD::Tn* mutants were similarly attenuated, although the Δ*fmt* mutant had a slightly higher, though not statistically significant, bacterial burden (Fig. 6).

## DISCUSSION

Folic acid metabolism represents a critical aspect of central metabolism, being required for the synthesis of purines, pyrimidines, some amino acids, and critically, formylated methionine (N-formylmethionine). In addition, folic acid metabolism is an established target for antimicrobial therapy. However, despite the wide application of antifolate antibiotics, the precise role of folic acid metabolism during bacterial pathogenesis has remained obscure. The goal of this study was to identify which specific folic acid-derived metabolites are required during infection of the facultative intracellular pathogen *L. monocytogenes*.

This study began with the identification of an *L. monocytogenes* mutant that formed very small plaques in tissue culture caused by disruption of *folD*, which encodes bifunctional methylenetetrahydrofolate dehydrogenase/methenyltetrahydrofolate cyclohydrolase (Fig. 1) (16). FolD is a bifunctional enzyme that plays a central role in the formation of most folic acid metabolites and is essential in many bacteria, although some bacteria, including *L. monocytogenes*, possess another enzyme, formyltetrahydrofolate synthetase (Fhs) that also catalyzes the formation of N10-formyl-tetrahydrofolate. The results of this study showed that *folD* mutants had significant defects during growth in tissue culture cells and a 2-log_10_ defect in the livers of mice. Fhs mutants had no apparent defects during growth in tissue culture cells or during infection of mice, but mutants lacking both FolD and Fhs were severely attenuated during infection of mice. Since Fhs uses formate as its substrate and *L. monocytogenes* only produces formate during anaerobic growth (44), bacteria may be experiencing low oxygen conditions *in vivo*, leading to the Fhs-dependent production of N10-formyl-tetrahydrofolate. *L. monocytogenes* may also benefit from having an alternative way of making N10-formyl-THF by Fhs during its natural route of infection, which requires growth in the low oxygen environment of the intestinal tract.

During a genetic screen looking for Δ*folD* suppressors, we found that loss of *purR*, the purine synthesis repressor, restored WT plaque formation in L2 cells, suggesting that *folD* mutants are defective in purine biosynthesis. There are numerous examples of bacterial purine auxotrophs having growth and virulence defects *in vivo* (45). For example, *Staphylococcus aureus* purine auxotrophs cannot replicate in human blood and serum and have defects in animal models (46, 47). Purine auxotrophs of *Bacillus anthracis* are dramatically attenuated in a murine infection model (48). Purine auxotrophic mutants of *Salmonella typhimurium* have a 4-log_10_ higher LD_50_ compared with WT (49, 50). In contrast to *Salmonella*, *L. monocytogenes* purine auxotrophic mutants are only moderately attenuated in terms of LD_50_ (51). The Δ*purEK* mutants generated in this study exhibited only an approximately 1.5-log_10_ defect in the livers of infected mice (Fig. 4C). Notably, this moderate defect suggests that *L. monocytogenes* can acquire purines from the host environment, although it remains to be investigated whether the defect of *L. monocytogenes* purine mutants is due to insufficient host purines during intracellular and/or extracellular growth. However, although Δ*purEK* mutants were attenuated, they were more than 100-fold more virulent than mutants lacking both FolD and Fhs in mice, indicating that purine deficiency is not the major defect when *L. monocytogenes* lacks N10-formyl-THF during infection.

In addition to purine biosynthesis, 1C metabolism mediated by folates integrates carbon units from serine, glycine, and sometimes formate to generate thymidine and fMet. In this study, we characterized *L. monocytogenes* mutants unable to synthesize thymidine (*thyA*), fMet (*fmt*), or folate precursor PABA (*pabBC*). Synthesis of dTMP by ThyA was essential for *L. monocytogenes* growth in BMMs (Fig. S5A) but dispensable during mouse infection (Fig. S5B). Strikingly, the *fmt* mutants led to the highest degree of attenuation *in vivo*, statistically identical to the Δ*pabBC* strain (Fig. 6), indicating that lack of fMet was the major defect of folate deficiency during infection. Previous studies demonstrated that antifolate antibiotics cause thymineless death of bacteria in nutrient-rich condition and inhibit bacteria growth by glycine and purine depletion in nutrient-limited conditions (52, 53). The observations of this study suggest that antifolate drugs also deplete fMet production during bacteria pathogenesis. However, our results do not eliminate the possible contribution of folates to serine and glycine generation during *L. monocytogenes* infection, since serine and glycine not only serve as carbon sources of folates but are also folate end-products if needed (6). Previous ^13^C-isotopologue profiling studies demonstrated that significant fractions (50%–100%) of bacterial amino acids, including serine and glycine, were from the host cell cytosol during intracellular growth (41, 54). We propose that the *in vivo* host environment provides some or all of a number of folate metabolites, including purines, pyrimidines, and amino acids but not fMet.

N-formylmethionine is a modified amino acid used by bacteria and mitochondrial proteins. The formylated methionine carried by initiator tRNA interacts with initiation factor 2 with a higher affinity than an unformylated methionine and thereby fMet facilitates high efficacy and fidelity of peptide translation in bacteria and mitochondria (19, 20, 35). Although fMet is important for *L. monocytogenes* infection, protein translation in *L. monocytogenes* is not strictly dependent on fMet. Mutants lacking *fmt* grew slower in brain heart infusion (BHI) media and were less attenuated in mouse spleens than livers. Similar to our findings in *L. monocytogenes*, fMet is not essential in many other bacteria, including *Escherichia coli*, *Pseudomonas aeruginosa*, *Mycobacterium smegmatis*, *Mycobacterium bovis, Staphylococcus aureus*, *Salmonella enterica*, and *Bacillus subtilis*, but loss of *fmt* often leads to slow growth and hypersensitivity to stress (34 – 39). The differences we observed *in vivo* indicate that the efficiency and fidelity in protein translation are more strictly important for *L. monocytogenes* in the liver than the spleen, although the nature of the differences is not clear. While *fmt* is not essential in many bacteria, it is predicted to be essential in *Streptococcus pneumonia* and *M. tuberculosis* (55 – 57). The stringency of fMet dependence in different bacteria under various conditions shall be taken into consideration in the development and evaluation of new antifolate antibiotics.

Although fMet is not essential in many bacteria, removal of the formyl-group from a nascent peptide by PDF is essential across bacterial species, making PDF an attractive target of antibiotics such as actinonin (58). The critical role of folates in fMet generation is also revealed in other bacteria, as mutants resistant to actinonin not only mapped to the *fmt* gene but also to *folD* and *glyA* (59, 60) (see Fig. 1). Although PDF removes fMet from most proteins during translation, some formyl-groups escape removal and are released as short peptides that are key targets of the innate immune system recognized by chemotactic receptors on neutrophils and monocytes, both critical immune effector cells required for constraining and resolving bacterial infections (61 – 66). Mice deficient in formylated peptide receptors are more susceptible to *L. monocytogenes* infection, probably due to the lack of a rapid wave of neutrophil infiltration that occurs early upon infection (67, 68). Therefore, another potential consequence of antifolate antibiotics is that by blocking production of fMet they also block the release of formylated peptides which would prevent detection of bacteria by phagocytic cells.

## MATERIALS AND METHODS

### Bacterial growth conditions

All *L. monocytogenes* strains used in this study were derived from WT strain 10403S (Table S3). Strains were propagated in filter-sterilized BHI medium (BD) at 37°C with shaking. When needed, bacterial culture media supplements were used at the following concentrations: streptomycin at 200  µg/mL, chloramphenicol at 7.5  µg/mL, erythromycin at 1 µg/mL, carbenicillin at 100 µg/mL, actinonin (MedChemExpress) at 100 µg/mL, and tetracycline at 2 µg/mL. The *Listeria* synthetic medium (LSM) was made using a previously described recipe with 20 amino acids added (69). When needed adenine was removed from the LSM recipe, or 10 mM sodium formate and 1 mM adenine were added to the medium. All reagents were purchased from Sigma-Aldrich unless specified.

Broth growth curves were performed with *L. monocytogenes* strains from overnight cultures grown at 37°C with agitation (220 rpm). BHI and LSM growth curves were started at an optical density (OD_600_) of 0.05. Growth was spectrophotometrically measured until saturated.

### Plasmid and strain construction

All strains used in this study are listed in Table S3. Plasmids were introduced into *L. monocytogenes* by conjugation, using a donor *E. coli* SM10 and a compatible *L. monocytogenes* strain. In-frame deletion of genes was performed using allelic exchange as previously described (70). The double mutants lacking both *folD* and *fhs* were generated by U153 phage transduction (71). Transductants were selected on erythromycin. The Δ*fmt* strain was selected in the presence of 100 µg/mL actinonin. The complemented strains were generated by integrating a pPL2 vector encoding genes under control of the p*Hyper* promoter (21) into mutant strain genomes and selecting for tetracycline-resistant transconjugants (72).

### Plaque assay

Plaque assays were performed as previously described (73). In brief, L2 fibroblasts and TIB73 hepatocytes were propagated in high-glucose Gibco Dulbecco’s modified Eagle medium (DMEM) (Thermo Fisher Scientific) plus 10% fetal bovine serum (FBS) (Avantor-Seradigm), 1 mM sodium pyruvate (Corning), and 2 mM L-glutamine (Corning) were plated 1.2 × 10^6^ cells/well in a six-well tissue-culture treated plate and incubated overnight. Prior to infection, TIB73 was washed twice with phosphate-buffered saline (PBS) and incubated in cell culture medium containing 0.1% FBS. *L. monocytogenes* strains were grown at 30°C overnight without agitation. Bacteria were diluted, 1:500 (for TIB73), 1:10,000 (for L2), in prewarmed media and overlaid on host cells. After 1 h, the cells were washed with PBS twice and overlayed with media plus 0.7% agarose and gentamicin at 10 µg/mL. Cells were stained with Neutral Red for at least 6 h prior to imaging 3-d post-infection. Plaque areas were measured using ImageJ software (74), collecting more than four plaques per strain per experiment.

### BMMs and intracellular growth curves

BMMs were prepared by collecting bone marrow from 8-wk-old female C57BL/6J mice (The Jackson Laboratory) and differentiated as previously described (75). All BMMs used in the experiments were cultured in DMEM with 20% FBS, 10% macrophage colony-stimulating factor, 1% L-glutamine, 1% sodium pyruvate, and 14 mM 2-mercaptoethanol (Thermo Fisher Scientific).

The intracellular growth curves were performed as previously described (75). Prior to infection, 3 × 10^6^ BMMs were seeded in 60-mm non-tissue culture-treated dishes (MIDSCI) containing 14 12-mm glass coverslips (Thermo Fischer Scientific) overnight. *L. monocytogenes* strains were grown at 30°C overnight without agitation. On the day of infection, bacteria were diluted in sterile PBS and infected BMMs at an MOI of 0.25. At 30-min post-infection, BMMs were washed twice with PBS. At 1-h post-infection, 50 µg/mL gentamicin was added to the cell culture media to prevent bacteria from growing extracellularly. Bacteria growth was enumerated by plating the average CFUs on three coverslips at each desired time point.

### Mouse infections

Eight-wk-old CD-1 mice (Charles River) were infected intravenously with 1 × 10^5^ CFUs in 200 µL of PBS. Animals were sacrificed at 48-h post-infection, and spleens and livers were harvested in 5 or 10 mL 0.1% IGEPAL CA-630 in water, respectively, and plated for enumeration of bacterial burdens. The virulence of ∆*inlB* ∆*actA* and ∆*inlB* ∆*actA folD::Tn* was determined by challenging 1 × 10^7^ CFUs per mouse.

### ∆*folD* suppressor screen

The ∆*folD* strain was mutagenized with *himar1* transposons as previously described (76, 77). The ∆*folD* libraries were cultured in BHI for 4 h before infecting L2 cells. Plaques visibly larger than those formed by ∆*folD* were picked and recovered in BHI then plaque again. The insertions of suppressor strains were transduced into the unmutagenized ∆*folD* parent strain, and the plaque assay was performed again to verify a single transposon insertion could repeat the increased plaque size. Transposon locations were determined using arbitrarily primed PCR as described previously (16).

### Disk diffusions

Antibiotic susceptibility was determined as previously described (78). In brief, 10^7^ bacteria were immobilized in 3 mL of top-agar (0.8% agar and 0.8% NaCl in water) and evenly distributed on 20-mL BHI-agar plate. Sterile filter paper disks were placed in the center of the agar plate, soaked with 10 µL actinonin stock solution (50 mg/mL in DMSO), and incubated overnight at 37°C. Clear zone areas were measured using ImageJ software (74).

### Statistical analysis

All statistical analyses were performed using GraphPad Prism version 9.2 for Windows, GraphPad Software, La Jolla, CA, USA, www.graphpad.com.

## SUPPLEMENTAL MATERIAL

Fig. S1 mbio.01074-23-s0001.pdf Lack of *folD* does not lead to defects in hepatocytes.

Fig. S2 mbio.01074-23-s0002.pdf Virulence in CD-1 mice of *L. monocytogenes* mutants lacking *folD* in a PrfA* background.

Fig. S3 mbio.01074-23-s0003.pdf Broth growth curve in *Listeria* synthetic media (LSM) supplemented with or without 10 mM sodium formate.

Fig. S4 mbio.01074-23-s0004.pdf Bacterial growth on *Listeria* synthetic media (LSM) agarose plates with or without 1 mM adenine added.

Fig. S5 mbio.01074-23-s0005.pdf Virulence of *L. monocytogenes* mutants lacking *thyA*.

Table S1 mbio.01074-23-s0006.pdf Doubling time of *L. monocytogenes* strains.

Table S2 mbio.01074-23-s0007.pdf Transposon mutations that suppress Δ*folD* plaque defect.

Table S3 mbio.01074-23-s0008.docx List of strains.

ASM does not own the copyrights to Supplemental Material that may be linked to, or accessed through, an article. The authors have granted ASM a non-exclusive, world-wide license to publish the Supplemental Material files. Please contact the corresponding author directly for reuse.
