# Supplementary material for: The major role of Listeria monocytogenes folic acid metabolism during infection is the generation of N-formylmethionine
Source: mBio. 2023 Sep 11;14(5):e01074-23. doi: 10.1128/mbio.01074-23 (PMC10653936; doi:10.1128/mbio.01074-23)
Supplement: Fig. S1 — Lack of folD does not lead to defects in hepatocytes. [file mbio.01074-23-s0001.pdf]

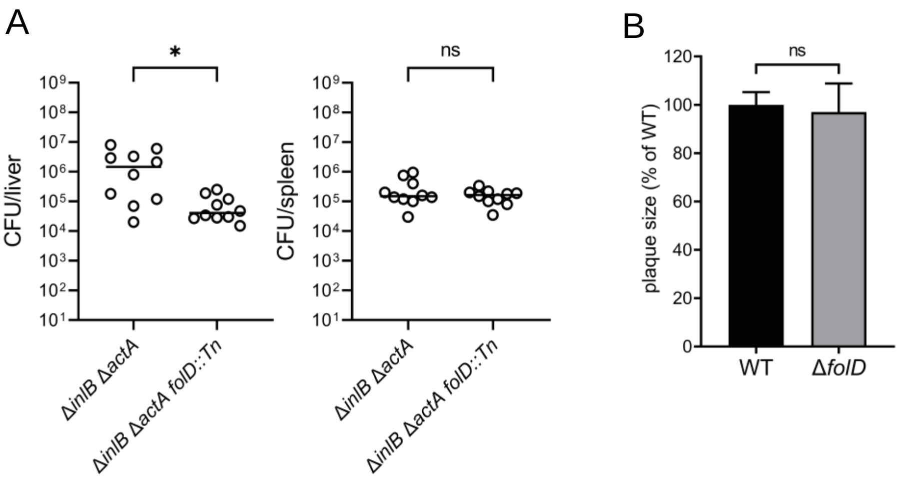

Figure S1. Lack of *folD* does not lead to defects in hepatocytes. (A) Bacteria growth in CD-1 mice infected intravenously with 10^7 CFUs per strain. Bacterial burdens in livers and spleens were measured 48-hour post infection by plating homogenized organs. Each circle represents an individual mouse. Lines present medians. (B) Plaque formation of *L. monocytogenes folD* mutants in murine hepatocyte cell line TIB-73 measured 3 days post-infection as a percentage of WT. Two biological replicates are combined with 5 mice per strain for each replicate. Student's *t*- test; ns, not significant; *$P < 0.05$.
