# Supplementary material for: The major role of Listeria monocytogenes folic acid metabolism during infection is the generation of N-formylmethionine
Source: mBio. 2023 Sep 11;14(5):e01074-23. doi: 10.1128/mbio.01074-23 (PMC10653936; doi:10.1128/mbio.01074-23)
Supplement: Fig. S2 — Virulence in CD-1 mice of L. monocytogenes mutants lacking folD in a PrfA* background. [file mbio.01074-23-s0002.pdf]

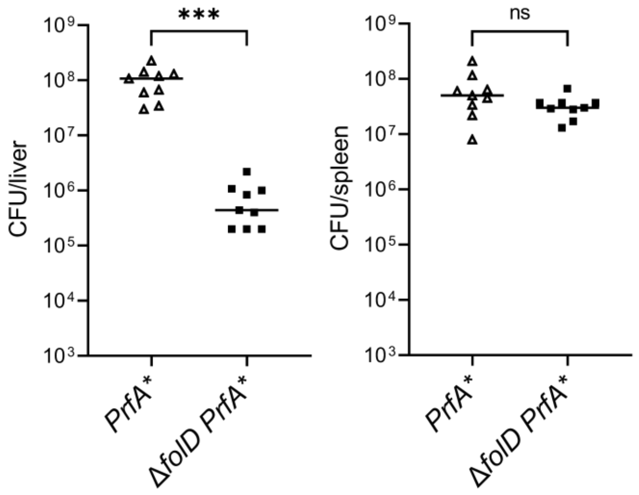

Figure S2. Virulence in CD-1 mice of L. monocytogenes mutants lacking *folD* in a *PrfA** background. Eight-week-old mice were infected intravenously with 1×10^5 CFUs of indicated strains. Bacterial burdens in livers and spleens were measured 48-hour post infection by plating homogenized organs. Each circle represents an individual mouse. Lines present medians. Two biological replicates are combined. Four or five mice for each group. Student's *t*- test; ns, not significant; ****P* < 0.001.
