# Supplementary material for: The major role of Listeria monocytogenes folic acid metabolism during infection is the generation of N-formylmethionine
Source: mBio. 2023 Sep 11;14(5):e01074-23. doi: 10.1128/mbio.01074-23 (PMC10653936; doi:10.1128/mbio.01074-23)
Supplement: Fig. S3 — Broth growth curve in Listeria synthetic media (LSM) supplemented with or without 10 mM sodium formate. [file mbio.01074-23-s0003.pdf]

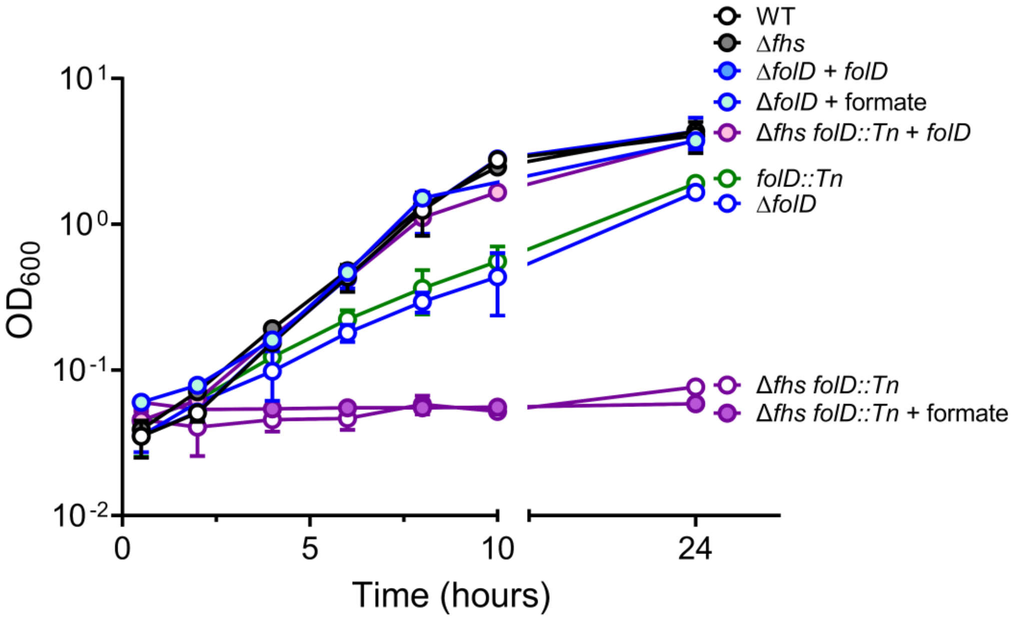

Figure S3. Broth growth curve in Listeria synthetic media (LSM) supplemented with or without 10 mM sodium formate. Strains were cultured at 37°C with agitation. Growth was measured spectrophotometrically. A representative result of two biological replicates is shown.
