# Supplementary material for: The major role of Listeria monocytogenes folic acid metabolism during infection is the generation of N-formylmethionine
Source: mBio. 2023 Sep 11;14(5):e01074-23. doi: 10.1128/mbio.01074-23 (PMC10653936; doi:10.1128/mbio.01074-23)
Supplement: Fig. S4 — Bacterial growth on Listeria synthetic media (LSM) agarose plates with or without 1 mM adenine added. [file mbio.01074-23-s0004.pdf]

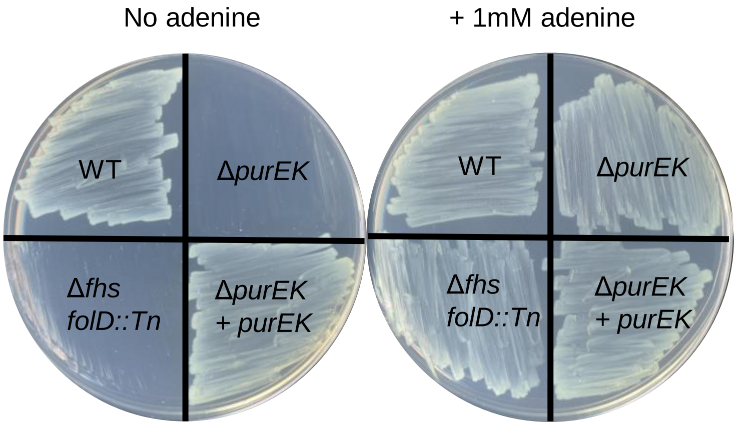

Figure S4. Bacterial growth on Listeria synthetic media (LSM) agarose plates with or without 1 mM adenine added. Purine auxotrophic mutant and its complemented strain were incubated at 37°C for two days.
