# Supplementary material for: The major role of Listeria monocytogenes folic acid metabolism during infection is the generation of N-formylmethionine
Source: mBio. 2023 Sep 11;14(5):e01074-23. doi: 10.1128/mbio.01074-23 (PMC10653936; doi:10.1128/mbio.01074-23)
Supplement: Fig. S5 — Virulence of L. monocytogenes mutants lacking thyA. [file mbio.01074-23-s0005.pdf]

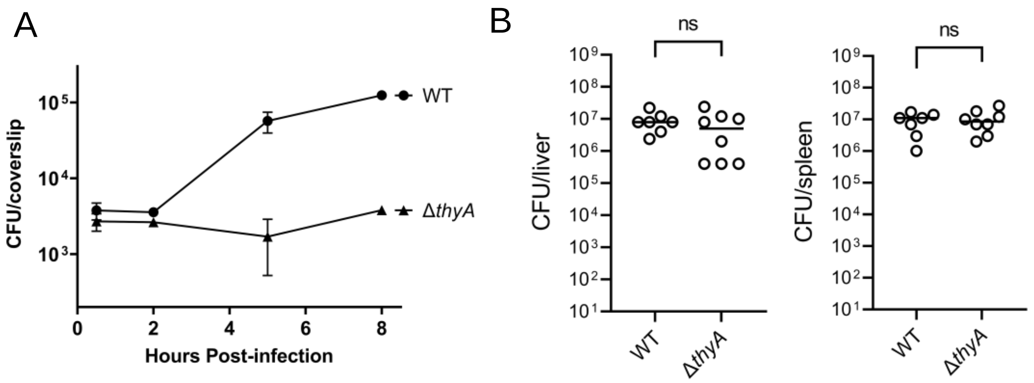

Figure S5. Virulence of *L. monocytogenes* mutants lacking *thyA*. (A) Intracellular growth in BMMs. BMMs were infected at an MOI of 0.25 for 30 minutes. Extracellular growth of bacteria was prevented by adding 50 µg/ml gentamicin at one-hour post infection. Growth was enumerated by plating the colony forming units (CFUs) at indicated time points. A representative result of two biological replicates is shown. (B) Eight-week-old mice were infected with 10^5 CFUs of indicated strain. Bacterial growth was measured at 2-day post infection. Two biological repeats are combined with a total of 7 or 8 mice for each strain. Student's *t*- test; ns, not significant.
