# Supplementary material for: The major role of Listeria monocytogenes folic acid metabolism during infection is the generation of N-formylmethionine
Source: mBio. 2023 Sep 11;14(5):e01074-23. doi: 10.1128/mbio.01074-23 (PMC10653936; doi:10.1128/mbio.01074-23)
Supplement: Table S1 — Doubling time of L. monocytogenes strains. [file mbio.01074-23-s0006.pdf]

Table S1. Doubling time of *L.monocytogenes* strains

Base medium	Strain	Supplementation	Doubling time[b]
BHI	10403S (WT)	-	41.4 ± 2.5
	Δ*folD*	-	41± 2.9
	Δ*fhs folD:Tn*	-	73.3 ± 2.0
	Δ*fmt*	-	72.6 ± 1.3
LSM[a]	WT	-	65.5 ± 4.8
	Δ*folD*	-	419.6 ± 56.4
	Δ*folD*	1 mM adenine	61.5 ± 1.7
	Δ*folD*	10 mM formate	60.8 ± 9.9
	Δ*fhs folD:Tn*	-	n/a[c]
	Δ*fhs folD:Tn*	1 mM adenine	175.0 ± 7.1
	Δ*fhs folD:Tn*	10 mM formate	n/a[c]
BMM	WT	-	42.5 ± 2.6
	Δ*fhs folD:Tn*	-	84.1 ± 13.6

[a]*Listeria* Synthetic Medium (LSM) with 20 aminio acids added.

[b]Doubling time presented as mean ± sd in minutes.

[c]Not available.

Doubling time of *L. monocytogenes* strains. Bacteria were grown in BHI or LSM broth cultured at 37°C with agitation. Growth was measured spectrophotometrically. The growth in BMMs were determined by plating the colony forming units (CFUs). The OD_{600} and CFUs during exponential phase were used to calculate doubling time by fitting non-linear growth curve equation using GraphPad Prism version 9.2.
