# Supplementary material for: The major role of Listeria monocytogenes folic acid metabolism during infection is the generation of N-formylmethionine
Source: mBio. 2023 Sep 11;14(5):e01074-23. doi: 10.1128/mbio.01074-23 (PMC10653936; doi:10.1128/mbio.01074-23)
Supplement: Table S2 — Transposon mutations that suppress ΔfolD plaque defect. [file mbio.01074-23-s0007.pdf]

Table S2. Transposon mutations that suppress Δ*folD* plaque defect

Gene name	Lmgr/lmo number	# Hits[a]	Annotation	Plaque size (%WT)[b]	
				WT[c]	Δ*folD*[c]
			10403S (WT)	100 ± 19.0	
			Δ*folD*	27.2 ± 4.4	
purR	02614/0192	3	*pur* operon repressor	95.8 ± 6.4	91.6 ± 11.3
aprt	01446/1524	1	adenine phosphoribosyltransferase	92.0 ± 12.6	50.5 ± 4.7
ade	02529/1742	1	adenine deaminase	97.2 ± 6.3	49.7 ± 5.9
pykA[e]	01397/1570	1	pyruvate kinase	60.5 ± 7.5	45.5 ± 4.8
	01205/2054	1	hypothetical protein	93.4 ± 8.2	45.7 ± 4.2
	00255/0573	1	AGZA family MFS transporter xanthine/uracil permease	99.8 ± 7.6	43.1 ± 5.2
	02408/0163	1	hypothetical protein, stage 0 sporulation protein yaaT	87.0 ± 10.2	42.3 ± 3.7
ndh2	02183/2638	5	NADH dehydrogenease	89.7 ± 5.3	43.1 ± 5.0

[a]Number of independent hits.

[b]Data represent the mean ± SD for three independent experiments.

[c]Genetic background of the transposon mutants..

[e]Transposon insertion was upstream of the *pykA* gene

Transposon mutations that suppress Δ*folD* plaque defect. Plaques visibly larger than those formed by Δ*folD* were picked. Transposon locations were determined using arbitrarily primed PCR. The insertions of suppressor strains were transduced into the unmutagenized Δ*folD* parent strain and the wild-type (WT) strain. The plaque assay was performed again to verify a single transposon insertion could repeat the increased plaque size.
