# Supplementary material for: The major role of Listeria monocytogenes folic acid metabolism during infection is the generation of N-formylmethionine
Source: mBio. 2023 Sep 11;14(5):e01074-23. doi: 10.1128/mbio.01074-23 (PMC10653936; doi:10.1128/mbio.01074-23)
Supplement: Table S3 — List of strains. [file mbio.01074-23-s0008.docx]

Table S3. List of strains

| **Stock** | **Background** | **Strain name** | **Plasmid** | **Reference** |
| --- | --- | --- | --- | --- |
| DP-E7508 | *E. coli* sm10 |  | pKSV7x.Δ*folD* | This study |
| DP-E7509 | *E. coli* sm10 |  | pPL2x.p*Hyper*.*folD* | This study |
| DP-E7510 | *E. coli* sm10 |  | pKSV7x.Δ*purR* | This study |
| DP-E7511 | *E. coli* sm10 |  | pKSV7x.Δ*fhs.s* | This study |
| DP-E7512 | *E. coli* sm10 |  | pKSV7x.Δ*fmt* | This study |
| DP-E7513 | *E. coli* sm10 |  | pPL2x.p*Hyper*.*fmt* | This study |
| DP-E7514 | *E. coli* sm10 |  | pKSV7x.Δ*purEK* | This study |
| DP-E7515 | *E. coli* sm10 |  | pPL2x.*purEpro.purEK* | This study |
| DP-L7273 | *L. monocytogenes* 10403S | *folD::Tn* |  | (1) |
| DP-L7516 | *L. monocytogenes* 10403S | *folD::Tn* + *folD* | pPL2x.p*Hyper*.*folD* | This study |
| DP-L7517 | *L. monocytogenes* 10403S | Δ*folD* |  | This study |
| DP-L7518 | *L. monocytogenes* 10403S | Δ*folD* *+ folD* | pPL2x.p*Hyper*.*folD* | This study |
| DP-L7519 | *L. monocytogenes* 10403S | Δ*fhs* |  | This study |
| DP-L7520 | *L. monocytogenes* 10403S | Δ*folD fhs::Tn* |  | This study |
| DP-L7521 | *L. monocytogenes* 10403S | Δ*fhs folD::Tn* |  | This study |
| DP-L7522 | *L. monocytogenes* 10403S | Δ*fhs folD::Tn* + *folD* | pPL2x.p*Hyper*.*folD* | This study |
| DP-L5451 | *L. monocytogenes* 10403S | PrfA* |  | (2) |
| DP-L7523 | *L. monocytogenes* 10403S | Δ*folD* PrfA* |  | This study |
| DP-L6054 | *L. monocytogenes* 10403S | ∆*inlB* ∆*actA* |  | (3) |
| DP-L7524 | *L. monocytogenes* 10403S | ∆*inlB* ∆*actA folD::Tn* |  | This study |
| DP-L7525 | *L. monocytogenes* 10403S | Δ*purR* |  | This study |
| DP-L7526 | *L. monocytogenes* 10403S | Δ*folD* Δ*purR* |  | This study |
| DP-L7527 | *L. monocytogenes* 10403S | Δ*folD fhs::Tn* Δ*purR* |  | This study |
| DP-L7528 | *L. monocytogenes* 10403S | Δ*folD aprt::Tn* |  | This study |
| DP-L7529 | *L. monocytogenes* 10403S | Δ*folD ade::Tn* |  | This study |
| DP-L7530 | *L. monocytogenes* 10403S | Δ*folD ndh2::Tn* |  | This study |
| DP-L7531 | *L. monocytogenes* 10403S | Δ*folD pykA::Tn* |  | This study |
| DP-L7532 | *L. monocytogenes* 10403S | Δ*folD lmo0163::Tn^a^* |  | This study |
| DP-L7533 | *L. monocytogenes* 10403S | Δ*folD lmo0573::Tn^b^* |  | This study |
| DP-L7534 | *L. monocytogenes* 10403S | Δ*folD lmo2054::Tn^c^* |  | This study |
| DP-L7535 | *L. monocytogenes* 10403S | Δ*fmt* |  | This study |
| DP-L7536 | *L. monocytogenes* 10403S | Δ*fmt* + *fmt* | pPL2x.p*Hyper*.*fmt* | This study |
| DP-L7537 | *L. monocytogenes* 10403S | Δ*purEK* |  | This study |
| DP-L7538 | *L. monocytogenes* 10403S | Δ*purEK* + *purEK* | pPL2x.*purEpro.purEK* | This study |
| DP-L7539 | *L. monocytogenes* 10403S | Δ*thyA* |  | (4) |
| DP-L7456 | *L. monocytogenes* 10403S | Δ*pabBC* |  | (5) |
| DP-L7540 | *L. monocytogenes* 10403S | Δ*folD purR::Tn* |  | This study |
| DP-L7541 | *L. monocytogenes* 10403S | *purR::Tn* |  | This study |
| DP-L7542 | *L. monocytogenes* 10403S | *aprt::Tn* |  | This study |
| DP-L7543 | *L. monocytogenes* 10403S | *ade::Tn* |  | This study |
| DP-L7544 | *L. monocytogenes* 10403S | *ndh2::Tn* |  | This study |
| DP-L7545 | *L. monocytogenes* 10403S | *pykA::Tn* |  | This study |
| DP-L7546 | *L. monocytogenes* 10403S | *lmo0163::Tn^a^* |  | This study |
| DP-L7547 | *L. monocytogenes* 10403S | *lmo0573::Tn^b^* |  | This study |
| DP-L7548 | *L. monocytogenes* 10403S | *lmo2054::Tn^c^* |  | This study |

^a^The corresponding gene tag in 10403S is LMRG_02408.

^b^The corresponding gene tag in 10403S is LMRG_00255.

^c^The corresponding gene tag in 10403S is LMRG_01205.

References

1. Anaya-Sanchez A, Feng Y, Berude JC, Portnoy DA. 2021. Detoxification of methylglyoxal by the glyoxalase system is required for glutathione availability and virulence activation in Listeria monocytogenes. PLoS Pathog 17::e1009819.

2. Miner MD, Porta GC, Freitag NE. 2008. Functional impact of mutational activation on the Listeria monocytogenes central virulence regulator PrfA. Microbiology 154::3579–3589.

3. Brocksted DG, Giedlin MA, Leong ML, Bahjat KS, Gao Y, Luckett W, Liu W, Cook DN, Portnoy DA, Dubensky TW. 2004. Listeria-based cancer vaccines that segregate immunogenicity from toxicity. Proc Natl Acad Sci U S A 101::13832–13837.

4. Tang Q, Precit MR, Thomason MK, Blanc SF, Ahmed-Qadri F, McFarland AP, Wolter DJ, Hoffman LR, Woodward JJ. 2022. Thymidine starvation promotes c-di-AMP-dependent inflammation during pathogenic bacterial infection. Cell Host Microbe 30::961-974.e6.

5. Zhang Y, Anaya-Sanchez A, Portnoy DA. 2022. para -Aminobenzoic Acid Biosynthesis Is Required for Listeria monocytogenes Growth and Pathogenesis. Infect Immun https:://doi.org/10.1128/iai.00207-22.
